# Neighborhood disadvantage, systemic inflammation, and tau pathology: a link between social determinants of health and Alzheimer's disease risk

**DOI:** 10.1002/alz70856_105926

**Published:** 2026-01-10

**Authors:** Brittany Butts, Enid Swatson, Jordan Watson, Chloe Park, Danielle D Verble, Kelly D. S. Likos, Christopher H Herring, Julia Kamara, Arin Watson, Sofie Ragins, Whitney Wharton

**Affiliations:** ^1^ Emory University, Atlanta, GA, USA

## Abstract

**Background:**

Neighborhood disadvantage, a key social determinant of health (SDOH), is associated with systemic inflammation and increased cardiometabolic risk, both of which contribute to Alzheimer's disease (AD) pathogenesis. While cardiovascular disease (CVD) is a known risk factor for cognitive decline, the role of systemic inflammation in linking neighborhood disadvantage to tau pathology remains unclear. Understanding these relationships may help identify modifiable targets for AD prevention, particularly among racially diverse populations at increased risk for both CVD and AD. This study examines the association between neighborhood disadvantage, inflammation, vascular stiffness, and tau biomarkers in middle‐aged and older adults living with CVD.

**Method:**

Participants (*N* = 40, age 66±8 years, 55% female, 38% Black adults) were enrolled in an ongoing cross‐sectional study. Neighborhood disadvantage was measured using the Area Deprivation Index (ADI). Serum inflammatory markers (C‐reactive protein [CRP] and triggering receptor expressed on myeloid cells‐1 [TREM‐1]), tau biomarkers (total tau [t‐tau] and phosphorylated tau [p‐tau217]), and vascular stiffness (pulse wave velocity [PWV]) were assessed.

**Result:**

Higher ADI was significantly associated with increased CRP (r=0.469, *p* = 0.002) and TREM‐1 (r=0.316, *p* = 0.047). Both CRP and TREM‐1 were positively correlated with PWV (r=0.334, *p* = 0.043; r=0.420, *p* = 0.0097). Higher levels of inflammation were associated with increased t‐tau (CRP: r=0.379, *p* = 0.016; TREM‐1: r=0.478, *p* = 0.002) and *p*‐tau217 (CRP: r=0.590, *p* <0.001). Black participants had higher ADI scores (*p* <0.05) but paradoxically lower *p*‐tau217 levels (*p* = 0.032) compared to white participants.

**Conclusion:**

These preliminary findings suggest that neighborhood disadvantage may contribute to AD risk through systemic inflammation and vascular dysfunction, leading to increased tau pathology. However, the observed racial differences in *p*‐tau217, with Black participants showing lower levels despite higher ADI scores and AD risk, align with prior studies. This raises questions about differences in tau processing, clearance, or non‐tau pathways of neurodegeneration. Future research should examine whether inflammation‐driven vascular dysfunction contributes disproportionately to cognitive decline in Black populations. Longitudinal studies are needed to assess whether interventions targeting inflammation and vascular health could mitigate AD risk and whether tau biomarkers accurately capture AD pathology across diverse populations. Understanding these mechanisms is crucial for ensuring equity in AD diagnosis and treatment.

